# Association between ambient air pollution and daily hospital admissions for ischemic stroke: A nationwide time-series analysis

**DOI:** 10.1371/journal.pmed.1002668

**Published:** 2018-10-04

**Authors:** Yaohua Tian, Hui Liu, Zuolin Zhao, Xiao Xiang, Man Li, Juan Juan, Jing Song, Yaying Cao, Xiaowen Wang, Libo Chen, Chen Wei, Yonghua Hu, Pei Gao

**Affiliations:** 1 Department of Epidemiology and Biostatistics, School of Public Health, Peking University, Beijing, China; 2 Medical Informatics Center, Peking University, Beijing, China; 3 Beijing HealthCom Data Technology Co. Ltd, Beijing, China; University of Wisconsin, Madison, UNITED STATES

## Abstract

**Background:**

Evidence of the short-term effects of ambient air pollution on the risk of ischemic stroke in low- and middle-income countries is limited and inconsistent. We aimed to examine the associations between air pollution and daily hospital admissions for ischemic stroke in China.

**Methods and findings:**

We identified hospital admissions for ischemic stroke in 2014–2016 from the national database covering up to 0.28 billion people who received Urban Employee Basic Medical Insurance (UEBMI) in China. We examined the associations between air pollution and daily ischemic stroke admission using a two-stage method. Poisson time-series regression models were firstly fitted to estimate the effects of air pollution in each city. Random-effects meta-analyses were then conducted to combine the estimates. Meta-regression models were applied to explore potential effect modifiers. More than 2 million hospital admissions for ischemic stroke were identified in 172 cities in China. In single-pollutant models, increases of 10 μg/m^3^ in particulate matter with aerodynamic diameter <2.5 μm (PM_2.5_), sulfur dioxide (SO_2_), nitrogen dioxide (NO_2_), and ozone (O_3_) and 1 mg/m^3^ in carbon monoxide (CO) concentrations were associated with 0.34% (95% confidence interval [CI], 0.20%–0.48%), 1.37% (1.05%–1.70%), 1.82% (1.45%–2.19%), 0.01% (−0.14%–0.16%), and 3.24% (2.05%–4.43%) increases in hospital admissions for ischemic stroke on the same day, respectively. SO_2_ and NO_2_ associations remained significant in two-pollutant models, but not PM_2.5_ and CO associations. The effect estimates were greater in cities with lower air pollutant levels and higher air temperatures, as well as in elderly subgroups. The main limitation of the present study was the unavailability of data on individual exposure to ambient air pollution.

**Conclusions:**

As the first national study in China to systematically examine the associations between short-term exposure to ambient air pollution and ischemic stroke, our findings indicate that transient increase in air pollution levels may increase the risk of ischemic stroke, which may have significant public health implications for the reduction of ischemic stroke burden in China.

## Abstract 2

Yonghua Hu and colleagues, in a large-scale analysis of 172 cities in China, reveal associations between short-term air pollution exposure and increased incidences of stroke.

## Author summary

**Why was this study done?:**

- Epidemiological studies have reported associations between short-term exposure to ambient air pollution and mortality and morbidity from ischemic stroke.
- Previous studies have been primarily conducted in high-income countries, and few research data at the country level have been generated in low- and middle-income countries, despite their much higher air pollution levels.

**What did the researchers do and find?:**

- We conducted a national time-series study using data on more than 2 million hospital admissions for ischemic stroke in 172 cities in China from 2014 to 2016.
- City-specific associations between ambient air pollution and hospital admissions for ischemic stroke were estimated with Poisson time-series regression models. Random-effects meta-analyses were used to obtain national and regional average associations.
- Our results indicated that short-term exposures to PM_2.5_, SO_2_, NO_2_, and CO were associated with increased hospital admissions for ischemic stroke. The associations were stronger in the elderly.

**What do these findings mean?:**

- To our knowledge, this is the first national study in China to systematically examine the associations between short-term exposure to ambient air pollution and ischemic stroke.
- Our findings strengthened the rationale for further limiting air pollution levels in low- and middle-income countries.

## Introduction

Stroke is a major public health concern that caused 6.5 million deaths and 113 million disability-adjusted life years worldwide in 2013 [1]. Epidemiological studies have examined the associations between short-term increases in ambient air pollution and risk of mortality and morbidity from ischemic stroke (and the predominant subtype of stroke), but the findings have been inconsistent [2–7]. According to the Global Burden of Disease Study 2013, air pollution accounts for more than a quarter of the stroke burden [8]. Compared with well-documented risk factors for ischemic stroke, such as smoking, poor diet, and physical inactivity [8], air pollution represents a potentially modifiable risk factor that is independent of changes in an individual’s behavior. Therefore, improving air quality has considerable public health implications for the reduction of ischemic stroke burden.

Previous studies on the acute effects of ambient air pollution on ischemic stroke have been primarily conducted in high-income countries, with only limited information from low- and middle-income countries, despite the much higher air pollution levels in these countries. A systematic review of studies on the associations between short-term exposure to air pollution and risk of stroke also indicated the scarcity of scientific evidence generated in low- or middle-income countries [7]. Moreover, the potential effects of particulate matter with aerodynamic diameter <2.5 μm (PM_2.5_) on ischemic stroke have been less investigated, especially in low- and middle-income countries, although PM_2.5_ has been widely considered as the predominant pollutant in the air [9]. Considering the substantial differences in the levels of air pollution, weather patterns, population susceptibility, and sociodemographic characteristics across different geographic regions, scientific data specific to low- and middle-income countries are needed.

As the largest low-middle–income country, China bears the greatest burden of ischemic stroke [10] and has also been experiencing the severest ambient air pollution in the world [11]. The adverse effects of air pollution on public health are of increasing concern in China, particularly in relation to haze days [12]. However, although several recent studies have examined the relations between air pollution and ischemic stroke morbidity risk in China, the studies were restricted to a single city or large hospitals. The results have been inconclusive, with some [13–16] but not other [17,18] studies finding evidence of a positive association. Therefore, the generalizability of the effect estimates from prior studies might be uncertain at the national level. In addition, these studies have limited ability to explore possible effect modifiers of the relations because of small numbers of study sites involved. To our knowledge, there have been no national studies on the acute effects of air pollution on ischemic stroke morbidity in China.

Since 2013, China has gradually introduced PM_2.5_ and ozone (O_3_) in the national air quality monitoring network and publicized real-time monitoring data. In addition, national morbidity data are available, along with the establishment of social health insurance schemes in China [19]. We therefore conducted a national study to examine the associations between short-term exposure to air pollution and daily hospital admissions for ischemic stroke in China during 2014–2016 under different circumstances.

## Materials and methods

### Study population

China achieved universal health insurance coverage in 2011, which now has three main insurance schemes. The Urban Employee Basic Medical Insurance (UEBMI) covers urban employees and retired employees. All employers in urban areas, including government agencies and institutions, state-owned enterprises, private businesses, social organizations, and other private entities and their employees (retirees included), are obliged to enroll in UEBMI [20]. The Urban Residence Basic Medical Insurance covers urban residents, including children, students, elderly people without previous employment, and unemployed people. The New Rural Cooperative Medical Scheme covers rural residents. Private medical insurance covers little in China and is generally supplementary on top of the basic schemes. The data on city-specific hospital admissions for ischemic stroke in our study for 2014–2016 were obtained from UEBMI, administered by China’s Ministry of Human Resources and Social Security. At the end of 2016, the database contained information on 0.28 billion people in 31 provincial administrative regions (except Tibet, Hong Kong, Macao, and Taiwan), representing more than 20% of the total population in Mainland China. We identified daily hospital admissions for ischemic stroke based on the information of primary discharge diagnosis. In addition, conducted medical examinations (yes or no, and financial cost), i.e., brain computed tomography or magnetic resonance imaging used in this study, were also recorded in the national insurance database. To decrease the impacts of diagnostic errors, admissions without brain computed tomography or magnetic resonance imaging examination were excluded from this analysis. Admissions under age 18 were too few and thus were also excluded. For each hospitalization, we extracted the date of admission, sex, and age. Because the data used for this study were collected for administrative purposes without any individual identifiers, this study was exempted from Institutional Review Board approval by the Ethics Committee of Peking University Health Science Center, Beijing, China. The need for informed consent was also waived by the Institutional Review Board.

### Air pollution and meteorological data

Data on PM_2.5_, sulfur dioxide (SO_2_), nitrogen dioxide (NO_2_), carbon monoxide (CO), and O_3_ for each city were obtained from the National Air Pollution Monitoring System. There are 1–17 monitors in each city. A series of standards or regulations have been made on the locations of monitors and the monitoring process of air pollutants by the Chinese government [21], ensuring that the monitoring measurements could reflect air pollution levels of the urban background [22,23]. The monitoring data have been extensively used as a proxy for population exposure to air pollution in China [23–25]. For each city, we obtained daily mean levels of PM_2.5_, SO_2_, NO_2_, and CO and maximum daily 8-hour mean O_3_ concentrations averaged across the monitors [24–26]. We also collected daily mean air temperature and relative humidity for each city from the China Meteorological Data Sharing Service System. In this study, we examined the effects of short-term exposure to air pollution on daily hospital admissions for ischemic stroke. Short-term exposure generally indicates exposure of air pollution up to a lag of 7 days, in line with previous studies [7].

### Statistical analysis

In this study, cities with data on both health insurance and air pollution available were included. We applied a commonly used two-stage approach to obtain regional and national average estimates of the associations between air pollution and daily hospital admissions for ischemic stroke [26,27]. This method and the model used in this study were designed before the analyses were conducted. The primary analysis was based on single-pollutant models [24,26,28,29]. In the first stage, a time-series analysis using Poisson regression in a generalized additive model was applied to estimate the associations in each city. Confounding covariates were incorporated in the model, including temperature, relative humidity, public holidays, and day of the week, which are predefined according to previously published studies [24,26,28,29]. We controlled for seasonality and time trends using a natural cubic spline with seven degrees of freedom (*df*) per year to exclude unmeasured time trends longer than 2 months in hospital admissions for ischemic stroke [24]. The selection of 7 *df* per year for calendar time was based on the parameter used in several recent large national studies in China [24,25,30,31]. We adjusted for the nonlinear and delayed effects of weather conditions on ischemic stroke admissions by fitting natural cubic splines with 3 *df* for the 3-day moving average air temperature and relative humidity [14]. We also incorporated indicator variables for public holidays and day of the week to adjust for the difference in the baseline hospital admission rates for each day. The model was as shown below:

$$\begin{array}{l} \text{Log}\left[\text{E}\left({\text{Y}}_{\text{t}}\right)\right]=\alpha +\beta \left(\text{air}\;\text{pollutants}\right)+\text{day}\;\text{of}\;\text{the}\;\text{week}+\text{public}\;\text{holidays}+ns\left(\text{calendar}\;\text{time},\;df=7/\text{per}\;\text{year}\right)\\ +ns\left(\text{temperature},\;df=3\right)+ns\left(\text{relative}\;\text{humidity},\;df=3\right) \end{array}$$

where E(Y_t_) is the expected count of admissions for ischemic stroke on day t; β represents the log-relative risk of ischemic stroke associated with a unit increase of air pollutant levels; *ns*() indicates natural cubic spline function; public holidays and day of the week were included in the model as indicator variables; and temperature and relative humidity indicate 3-day moving average air temperature and relative humidity, respectively. We used same-day air pollutant concentrations (lag 0) in our main analyses, because it often produced the largest effect estimate in previous studies [5,7,28]. Single lags of 1 and 2 days were also conducted to explore the lag pattern in the effects. In the second stage, we conducted random-effects meta-analyses to pool the city-specific results for regional- or national-level estimates [26,27].

Subgroup analyses were performed stratified by individual characteristics, including sex and age (18–64, 65–74, and ≥75 years). Considering the substantial variations in air pollution levels, meteorological conditions, and topography between southern and northern China, defined by the Huai River–Qinling Mountains line [32,33], a stratified analysis by geographical region was also conducted [24,26]. We compared the stratified models using a z-test [34]. We also evaluated whether the associations between air pollution and daily ischemic stroke admission differed by city-level characteristics, including cities’ mean air pollutant levels, ambient temperatures, relative humidity, and gross domestic product (GDP) per capita using meta-regression models [24,26]. Data on GDP per capita were collected from city statistical yearbooks.

Several sensitivity analyses were conducted to assess the robustness of the results: (1) using different subsets of cities with 2- or 3-year data; (2) using different *df* values (6–12 per year) for time trend, temperature (4–6), and relative humidity (4–6); (3) two-pollutant models if an individual pollutant showed a statistically significant association with ischemic stroke admission (*P* < 0.05).

All results were reported as percentage changes and 95% confidence intervals (CIs) in daily hospital admissions for ischemic stroke, in association with increases of 10 μg/m^3^ in PM_2.5_, SO_2_, NO_2_, and O_3_ and 1 mg/m^3^ in CO exposures. To allow for comparison of the size of effects across the several pollutants, we also reported estimated effects in interquartile range (IQR) increments. All first-stage analyses were done using R, version 3.2.2 (R Foundation for Statistical Computing, Vienna, Austria). Meta-analyses were conducted in Stata statistical software, version 12 (StataCorp, College Station, TX).

## Results

**Fig 1:**
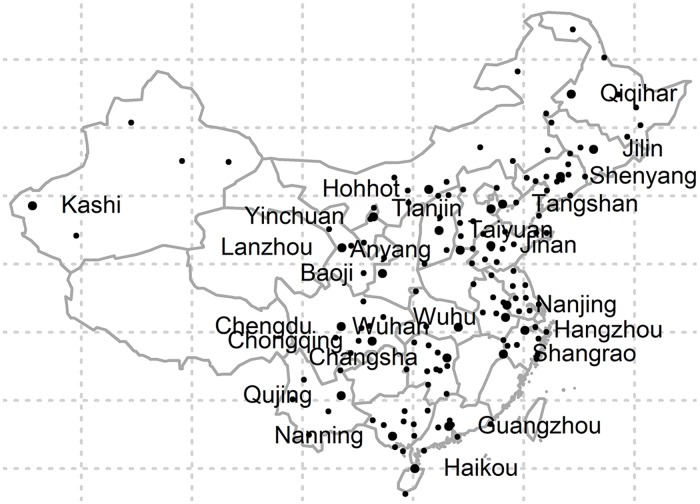
Locations of the 172 Chinese cities in this study. The most populous city in each province is marked with its name.

A total of 2,032,667 hospital admissions for ischemic stroke from 172 cities in China formed the basis of this study. Of the 172 cities, 86 cities were located in south region and 86 cities were in the north. The locations of the 172 cities are shown in Fig 1. Overall, there were 65.0% male patients and 35.5% patients aged ≥75 years. The sex distribution of ischemic stroke patients in the south versus north regions was similar, while the proportion of elderly patients was higher in southern China (Table 1). Table 2 presents the summary statistics of air pollutants and weather conditions from citywide averages of the monitors in the 172 Chinese cities between 2014 and 2016. The average annual means (standard deviation [SD]) of air pollutants were 51.5 (21.6) μg/m^3^ for PM_2.5_, 27.9 (16.5) μg/m^3^ for SO_2_, 31.0 (10.8) μg/m^3^ for NO_2_, 1.07 (0.35) mg/m^3^ for CO, and 86.1 (13.6) μg/m^3^ for O_3_, respectively. On average, cities in northern China have higher air pollutant levels and lower air temperatures and relative humidity. The levels of PM_2.5_, SO_2_, NO_2_, and CO were positively correlated with each other (correlation coefficient = 0.52–0.64, *P* < 0.001), while O_3_ was negatively correlated with other air pollutants (Table 3).

**Table 1:** Demographic characteristics of hospital admissions for ischemic stroke in 172 cities in China, 2014–2016.

Variable	Overall	Southern China	Northern China
Number of cities	172	86	86
Total	2,032,667	839,266	1,193,401
Sex			
Male (%)	1,320,444 (65.0)	521,504 (62.1)	798,940 (68.0)
Female (%)	712,223 (35.0)	317,762 (37.9)	394,461 (32.0)
Age (years)			
18–64 (%)	656,819 (33.3)	214,887 (26.3)	441,931 (38.2)
65–74 (%)	616,339 (31.2)	250,744 (30.7)	365,595 (31.6)
≥75 (%)	702,065 (35.5)	351,654 (43.0)	350,411 (30.3)

**Table 2:** Summary statistics† for air pollutant concentrations and meteorological variables in 172 cities in China, 2014–2016.

Variable	Mean ± SD	Minimum	Percentile			Maximum	IQR
			25th	50th	75th		
PM_2.5_ (μg/m^3^)	51.5 ± 21.6	15.7	37.5	49.6	59.7	122.3	22.2
Southern China	46.5 ± 13.7	15.7	35.8	47.8	57.7	78.9	21.9
Northern China	56.6 ± 26.4	16.7	38.8	51.4	70.3	122.3	31.5
PM_10_ (μg/m^3^)	91.8 ± 61.3	28.0	65.3	84.5	102.0	186.0	36.7
Southern China	72.3 ± 19.0	28.0	56.0	73.3	86.0	113.6	30.0
Northern China	110.2 ± 79.3	40.9	72.2	94.4	127.2	186.0	50.0
SO_2_ (μg/m^3^)	27.9 ± 16.5	6.1	17.0	23.0	34.5	78.3	17.5
Southern China	19.3 ± 7.1	6.1	13.9	19.9	23.4	40.6	9.5
Northern China	35.9 ± 18.7	6.7	19.3	33.1	50.1	78.3	30.8
NO_2_ (μg/m^3^)	31.0 ± 10.8	10.0	22.4	30.9	37.7	59.7	15.3
Southern China	29.7 ± 10.5	10.0	21.0	29.9	36.7	51.9	15.7
Northern China	32.2 ± 11.0	12.0	22.9	33.0	40.3	59.7	17.4
CO (mg/m^3^)	1.07 ± 0.35	0.39	0.85	1.00	1.26	2.24	0.41
Southern China	0.95 ± 0.21	0.39	0.82	0.95	1.06	1.51	0.24
Northern China	1.18 ± 0.42	0.47	0.88	1.14	1.44	2.24	0.56
O_3_ (μg/m^3^)	86.1 ± 13.6	51.4	77.5	86.7	96.2	118.0	18.7
Southern China	84.8 ± 12.5	51.4	77.5	83.6	93.7	109.0	16.2
Northern China	87.2 ± 14.6	55.2	78.0	88.4	97.1	118.0	19.1
Temperature (°C)	14.3 ± 5.4	0.3	10.6	14.9	17.8	24.9	7.2
Southern China	18.4 ± 2.9	10.0	16.6	17.8	20.7	24.9	4.1
Northern China	10.1 ± 3.8	0.3	7.5	10.7	13.3	16.9	5.8
Relative humidity (%)	68.7 ± 13.4	35.8	58.9	69.2	78.4	83.8	19.5
Southern China	79.0 ± 9.0	60.4	74.7	78.4	81.1	83.8	6.4
Northern China	58.4 ± 8.0	35.8	53.0	58.9	64.1	78.0	11.1

^†^Statistics were generated from citywide averages of the monitors.

Abbreviation: IQR, interquartile range.

**Table 3:** Spearman correlation coefficients among the exposure variables in 172 cities in China, 2014–2016.

Variables	PM_2.5_	PM_10_	SO_2_	NO_2_	CO	O_3_	Temp	RH
PM_2.5_	1.00	0.90^†^	0.57^†^	0.64^†^	0.60^†^	−0.15^†^	−0.25^†^	−0.09^†^
PM_10_	―	1.00^†^	0.61^†^	0.63^†^	0.56^†^	0.05^†^	−0.26^†^	−0.29^†^
SO_2_	―	―	1.00	0.57^†^	0.52^†^	−0.09^†^	−0.38^†^	−0.34^†^
NO_2_	―	―	―	1.00	0.53^†^	−0.13^†^	−0.30^†^	−0.12^†^
CO	―	―	―	―	1.00	−0.21^†^	−0.28^†^	−0.04^†^
O_3_	―	―	―	―	―	1.00	0.53^†^	−0.22^†^
Temp	―	―	―	―	―	―	1.00	0.25^†^
RH	―	―	―	―	―	―	―	1.00

^†^*P* < 0.01.

Abbreviations: RH, relative humidity; Temp, temperature.

**Fig 2:**
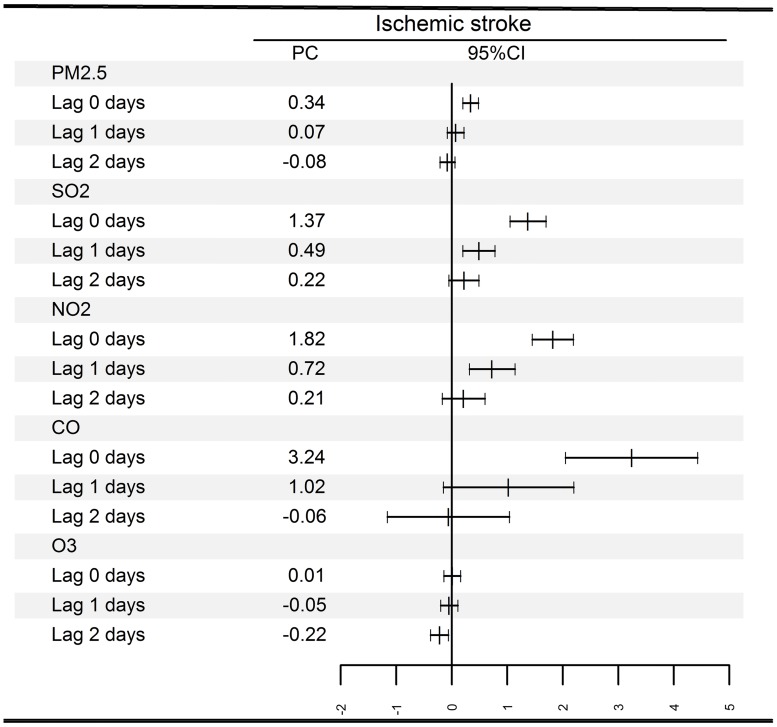
PC and 95% CI in daily hospital admissions for ischemic stroke associated with increases of 10 μg/m^3^ in PM_2.5_, SO_2_, NO_2_, and O_3_ and 1 mg/m^3^ in CO concentrations at different lag days. CI, confidence interval; PC, percentage change.

Fig 2 presents the national average estimates of the associations between air pollution and hospital admissions for ischemic stroke for different lag structures. All analyzed air pollutants, with the exception of O_3_, showed consistent significant associations at lag 0 day. Increases of 10 μg/m^3^ in concurrent day PM_2.5_, SO_2_, NO_2_, and O_3_ and 1 mg/m^3^ in CO exposure were associated with 0.34% (95% CI, 0.20%–0.48%), 1.37% (95% CI, 1.05%–1.70%), 1.82% (95% CI, 1.45%–2.19%), 0.01% (95% CI, −0.14%–0.16%), and 3.24% (95% CI, 2.05%–4.43%) increases in hospital admissions for ischemic stroke, respectively. The estimated effects in IQR increments are also presented in S1 Table.

**Fig 3:**
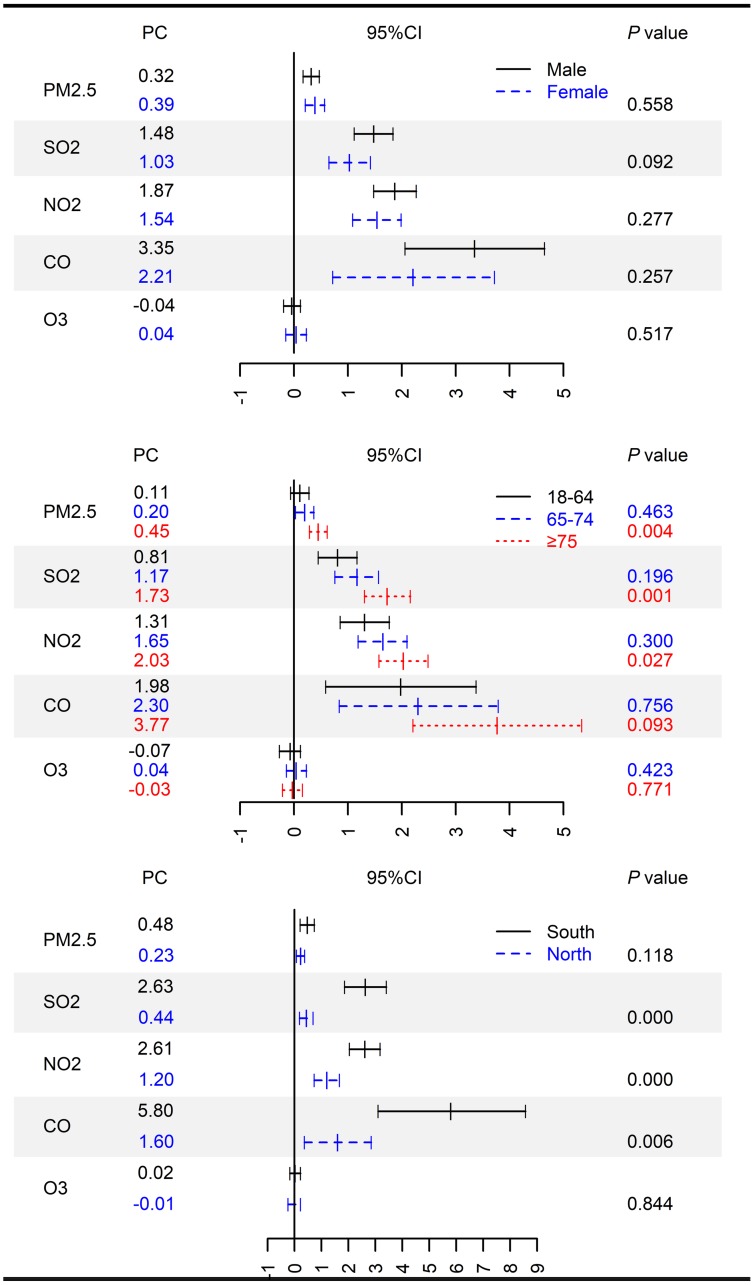
PC and 95% CI in daily hospital admissions for ischemic stroke associated with increases of 10 μg/m^3^ in PM_2.5_, SO_2_, NO_2_, and O_3_ and 1 mg/m^3^ in CO concentrations (lag 0), classified by sex, age, and region. *P* value obtained from z-test for the difference between the two risk estimates derived from stratified analysis. CI, confidence interval; PC, percentage change.

Fig 3 shows the associations between air pollutant levels (lag 0) and ischemic stroke admission, stratified by geographical region, sex, and age. The associations varied by geographical region and age. The associations were generally stronger in the south region, and also in patients aged ≥75 years. We did not observe evidence for effect modification by sex (*P* > 0.05). Table 4 shows the results from meta-regression analyses. Generally, the acute effects of air pollutants on ischemic stroke were greater in cities with lower air pollutant levels and higher air temperatures. We found no evidence for effect modification by relative humidity and GDP per capita.

**Table 4:** Relations between air pollutant effects and city-specific characteristics.

Variables	Percentage change	95% confidence interval	*P*
PM_2.5_			
PM_2.5_ (10 μg/m^3^)	−0.065	−0.151–0.022	0.143
Temperature (°C)	0.011	0.001–0.021	0.047
Relative humidity (%)	0.002	−0.012–0.016	0.741
GDP per capita	−0.044	−0.130–0.043	0.318
SO_2_			
SO_2_ (10 μg/m^3^)	−0.398	−0.708–−0.087	0.012
Temperature (°C)	0.196	0.075–0.316	0.002
Relative humidity (%)	0.066	0.025–0.016	0.002
GDP per capita	0.054	−0.200–0.308	0.676
NO_2_			
NO_2_ (10 μg/m^3^)	−0.487	−1.057–0.085	0.094
Temperature (°C)	0.176	0.056–0.296	0.004
Relative humidity (%)	0.023	−0.021–0.067	0.298
GDP per capita	−0.131	−0.390–0.129	0.320
CO			
CO (1 mg/m^3^)	−4.714	−8.926–−0.307	0.036
Temperature (°C)	0.309	−0.065–0.685	0.105
Relative humidity (%)	0.087	−0.046–0.222	0.199
GDP per capita	−0.138	−0.958–0.688	0.741

Abbreviation: GDP, gross domestic product.

Table 5 shows the results of two-pollutant models. The associations of ischemic stroke with SO_2_ and NO_2_ remained statistically significant when controlling for the effects of other air pollutants. However, the estimated effects of PM_2.5_ and CO exposure turned out to be statistically insignificant after adjustment of SO_2_ and NO_2_. Sensitivity analyses were present in S2 Table. Pooled significant associations between air pollution and daily hospital admissions for ischemic stroke were consistently observed across all cities, cities with 2-year data, and cities with 3-year data. The use of alternative *df* value for time trend (6–12 per year), temperature (4–6), and relative humidity (4–6) did not substantially affect the effect estimates of the associations between air pollution and daily hospital admissions for ischemic stroke.

**Table 5:** Associations of air pollution with hospital admissions for ischemic stroke in two-pollutant models.

Variable	PM_2.5_	SO_2_	NO_2_	CO
Adjusted for PM_2.5_	―	1.45 (1.07–1.83)	2.26 (1.80–2.71)	2.58 (0.95–4.24)
Adjusted for SO_2_	0.06 (−0.10–0.22)	―	1.61 (1.11–2.10)	1.17 (−0.27–2.62)
Adjusted for NO_2_	−0.17 (−0.34–0.01)	0.60 (0.19–1.02)	―	−0.64 (−2.29–1.05)
Adjusted for CO	0.17 (−0.02–0.37)	1.41 (1.01–1.81)	2.27 (1.75–2.80)	―

## Discussion

To the best of our knowledge, this is the first national study in China to systematically examine the associations between short-term exposure to ambient air pollution and ischemic stroke. Overall, our study suggested that transient increases in PM_2.5_, SO_2_, NO_2_, and CO were significantly associated with increased hospital admissions for ischemic stroke in 172 Chinese cities in single-pollutant models. The associations of SO_2_ and NO_2_ remained significant in two-pollutant models, but not PM_2.5_ and CO associations. The associations were stronger in the elderly. The associations varied with several city-level characteristics, including geographical region, air pollutant levels, and air temperatures. Our findings contributed to the limited evidence regarding the acute effects of air pollution on ischemic stroke in low- and middle-income countries.

In contrast to prior Chinese studies using morbidity data obtained from a single city or large hospitals, we analyzed the national data under a systematically consistent framework, thus significantly decreasing the impacts of publication bias as well as having greater statistical power to detect the associations of air pollution with ischemic stroke. We included 172 cities in China involving great diversity in air pollutant levels, geography, weather conditions, and socioeconomic status, which enabled us to explore the potential effect modification by these characteristics. Therefore, our study would yield more representative and stable effect estimates of the short-term associations between air pollution and ischemic stroke admission than prior studies offered.

All analyzed air pollutants, with the exception of O_3_, were significantly associated with increased ischemic stroke admissions in single-pollutant models in this study. A majority of previously published epidemiological studies were from high-income countries, yielding inconclusive results on this issue. For example, Wellenius and colleagues [35] estimated the short-term effects of air pollution on hospital admissions for ischemic stroke among Medicare beneficiaries (aged ≥65 years) residing in nine United States counties. IQR increases in same-day particulate matter with aerodynamic diameter <10 μm (PM_10_) (22.96 μg/m^3^), CO (0.71 parts per million [ppm]), NO_2_ (11.93 parts per billion [ppb] μg/m^3^), and SO_2_ (6.96 ppb) concentrations were associated with 1.03%, 2.83%, 2.94%, and 1.35% increases in ischemic stroke admissions, respectively. However, no association was found between short-term exposure to air pollution and stroke in eight French cities [36]. Similarly, a case-crossover study done in seven Australian and New Zealand cities failed to observe significant associations of air pollutant levels with stroke in elderly people [37]. In a meta-analysis of studies on the acute effects of air pollution on stroke, Shah and colleagues [7] estimated that the excess changes in daily hospital admissions for stroke associated with per 10 μg/m^3^ increment of PM_2.5_; 10 ppb increment of SO_2_, NO_2_, and O_3_; and 1 ppm increment of CO were 1.1% (95% CI, 1.0%–1.2%), 1.6% (95% CI, 0.4%–2.8%), 1.2% (95% CI, 0.5%–1.8%), 0.1% (95% CI, 0%–0.2%), and 1.1% (95% CI, −0.1%–2.3%), respectively. The inconsistency of the results on the associations between air pollution and stroke might be attributable to variations in air pollution levels, outcome definitions, weather conditions, population susceptibility, and sociodemographic characteristics across studies.

In two-pollutant models, the associations of ischemic stroke with SO_2_ and NO_2_ remained significant when controlling for the effects of other air pollutants. However, the estimated effects of PM_2.5_ and CO exposure turned out to be statistically insignificant after adjustment of SO_2_ and NO_2_. Our results were consistent with several multicenter studies. For example, two recent studies done in 272 Chinese cities reported significant effects of SO_2_ and NO_2_ on all-cause and cardiovascular mortality after controlling for PM_2.5_ and CO [31,38]. Liu and colleagues reported a significant association between CO and stroke mortality in single-pollutant model, but it became nonsignificant after adjustment of SO_2_ and NO_2_ [30]. A recent multi-city study done in 26 Chinese cities reported significant short-term associations between PM_10_, SO_2_, NO, and CO and hospital admissions for ischemic stroke in single-pollutant models. Similarly, in two-pollutant models, the associations of ischemic stroke with SO_2_ and NO_2_ remained stable and significant after adjusting for PM_10_ and CO, while the associations for PM_10_ and CO became nonsignificant when controlling for SO_2_ and NO_2_ [39]. NO_2_ generally serves as a surrogate measure for vehicular pollution because of its close association with vehicle exhaust emissions [40]. SO_2_ is largely from combustion of sulfur-containing fuels such as coal and oil. In China, a substantial proportion of PM_2.5_ originates from vehicle exhaust emissions [41]. The attenuation of the effect of PM_2.5_ can be interpreted by either that the PM_2.5_ effect may be confounded by other air pollutants or by the collinearity in the regression model, as suggested by a vast amount of literature [29,42], although the CIs of the effect estimations were not remarkably inflated in the two-pollutant model in our study.

The identification of potentially sensitive subpopulations has significant implications for scientific and public health purposes, as it may provide new insights into the mechanisms and help to target certain subgroups that need to reduce personal exposure during hazardous pollution days. In this study, we found that the effect estimates of air pollutants for the ≥75 age group were higher than for the 18–64 age group. It is now generally believed that elderly subjects are at increased risk of cardiovascular events associated with short-term exposure to air pollution [43].

Few studies have explored city-level characteristics in the relations between air pollution and ischemic stroke, possibly because of the small number of study sites in prior studies. In this nationwide study, we found smaller effects of air pollutants in cities with higher air pollutant levels. Similarly, several recent studies done in China reported weaker acute effects of air pollution on mortality risk in cities with higher air pollutant levels [24,26]. The relatively smaller effect estimates at higher air pollutant levels might be related to “harvesting effect” in that people who are vulnerable to air pollution exposure might have developed ischemic stroke symptoms and gone to hospitals before air pollution reached a fairly high level [44]. In addition, we found increased risk of ischemic stroke associated with higher air temperatures. Previous studies have demonstrated that high temperatures could enhance acute effects of air pollution on mortality risk [24,45].

Moreover, we explored the variation of risks between southern and northern China, and observed higher effect estimates in the south region, which is consistent with the reported higher mortality effects associated with air pollution in the south region [24,26]. There are several possible explanations for this regional variation in the magnitude of effect estimates. First, differences in demographic characteristics of ischemic stroke patients may contribute to the regional heterogeneity. For example, the south region has a remarkably higher proportion of patients aged ≥75 years, who were found to be more susceptible to adverse effects of air pollution in our study. Second, air pollution is a heterogeneous, complex mixture consisting of various compounds from multiple sources. The effects of each component or source on stroke remain unclear [43]. Because of the winter heating policy, cities located in the north region generally have a higher proportion of crustal components and materials in relation to biomass burning [46]. The differences in the composition and sources of air pollution between the south and north regions may be responsible for the spatial heterogeneity of effect estimates. Third, the substantial differences in the meteorological conditions between southern and northern China may be another possible explanation. In this study, the south region has substantially higher air temperature, which could enhance effects of air pollution on ischemic stroke, as shown in the meta-regression analysis (Table 4).

To further explore the variables contributing to the regional variation in the magnitude of effect estimates, an analysis on the association between temperature and ischemic stroke was conducted, showing that a 1 °C increase in the same-day temperature was associated with a 0.44% (0.29%–0.58%) increase in hospital admissions for ischemic stroke after controlling for calendar time, relative humidity, public holidays, and day of the week. The associations between air pollution and ischemic stroke were more evident in the warm season, compared with those in the cool season (S3 Table). In addition, we divided the 172 cities into four groups based on their annual average temperatures and air pollutant levels. High temperature could consistently enhance the effects of air pollution in both low- and high-polluted areas, while the positive effect modification of long-term air pollutant levels were less consistent (S4 Table). These findings indicate that high temperature might exert greater modifying effects on the associations than long-term air pollution levels.

This study has several potential limitations. First, the use of ambient air pollutant levels as a proxy for personal measures is expected to result in exposure measurement error, although this misclassification is expected to bias the risk estimates downward [47]. Second, the impacts of potential misclassification of ischemic stroke resulting from diagnostic error should be considered when interpreting the results. However, this error is unlikely related to air pollutant levels and is expected to reduce the precision of the estimates and bias the risk estimates downward [35]. Third, we linked air pollutant levels to ischemic stroke events based on date of admission rather than on date of symptom onset, which would cause non-differential error in exposure measurement and bias of the effect estimates toward null [47]. Finally, although one-stage and two-stage methods usually generate very similar results [48], the first-step variances in a two-stage analysis are often not exactly those implied in a one-stage method, which may further lead to the issue related to propagating the uncertainty and the correlation structure. However, computational problems in a one-stage method might be formidable in large data sets [49].

In conclusion, we found that short-term exposure to air pollution was associated with increased hospital admission for ischemic stroke in China. Our study also provided evidence that the acute effects of air pollution on ischemic stroke were modified by certain city-level characteristics.

## Supporting information

S1 Table PC and 95% CI in daily hospital admissions for ischemic stroke associated with IQR increases in same-day PM_2.5_ (22.2 μg/m^3^), SO_2_ (17.5 μg/m^3^), NO_2_ (15.3 μg/m^3^), CO (0.41 mg/m^3^), and O_3_ (18.7 μg/m^3^) concentrations. CI, confidence interval; IQR, interquartile range; PC, percentage change.
(DOCX)

S2 Table Results of sensitivity analyses on the associations between air pollution (lag 0) and hospital admissions for ischemic stroke in 172 cities in China, 2014–2016. (DOCX)

S3 Table PC and 95% CI in daily hospital admissions for ischemic stroke associated with increases of 10 μg/m^3^ in PM_2.5_, SO_2_, NO_2_, and O_3_ and 1 mg/m^3^ in CO concentrations (lag 0), classified by season. CI, confidence interval; PC, percentage change.
(DOCX)

S4 Table PC and 95% CI in daily hospital admissions for ischemic stroke associated with increases of 10 μg/m^3^ in PM_2.5_, SO_2_, NO_2_, and O_3_ and 1 mg/m^3^ in CO concentrations (lag 0), classified by city-specific annual average temperatures and air pollutant levels. CI, confidence interval; PC, percentage change.
(DOCX)

S1 STROBE Checklist (DOC)

## Abbreviations

CI: confidence interval
CO: carbon monoxide
*df*: degree of freedom
GDP: gross domestic product
IQR: interquartile range
NO_2_: nitrogen dioxide
O_3_: ozone
PM_10_: particulate matter with aerodynamic diameter <10 μm
PM_2.5_: particulate matter with aerodynamic diameter <2.5 μm
ppb: parts per billion
ppm: parts per million
SO_2_: sulfur dioxide
UEBMI: Urban Employee Basic Medical Insurance

## References

[1] Feigin VL, Norrving B, Mensah GA. Global Burden of Stroke. Circ Res. 2017;120(3):439–48. 10.1161/CIRCRESAHA.116.308413 28154096

[2] Hong YC, Lee JT, Kim H, Ha EH, Schwartz J, Christiani DC. Effects of air pollutants on acute stroke mortality. Environ Health Perspect. 2002;110(2):187–91. 10.1289/ehp.02110187 11836148 PMC1240734

[3] Chan CC, Chuang KJ, Chien LC, Chen WJ, Chang WT. Urban air pollution and emergency admissions for cerebrovascular diseases in Taipei, Taiwan. Eur Heart J. 2006;27(10):1238–44. 10.1093/eurheartj/ehi835 16537554

[4] Lisabeth LD, Escobar JD, Dvonch JT, Sanchez BN, Majersik JJ, Brown DL, et al Ambient air pollution and risk for ischemic stroke and transient ischemic attack. Ann Neurol. 2008;64(1):53–9. 10.1002/ana.21403 18508356 PMC2788298

[5] Wellenius GA, Burger MR, Coull BA, Schwartz J, Suh HH, Koutrakis P, et al Ambient air pollution and the risk of acute ischemic stroke. Arch Intern Med. 2012;172(3):229–34. 10.1001/archinternmed.2011.732 22332153 PMC3639313

[6] Chung JW, Bang OY, Ahn K, Park SS, Park TH, Kim JG, et al Air Pollution Is Associated With Ischemic Stroke via Cardiogenic Embolism. Stroke. 2017;48(1):17–23. 27899751 10.1161/STROKEAHA.116.015428

[7] Shah AS, Lee KK, McAllister DA, Hunter A, Nair H, Whiteley W, et al Short term exposure to air pollution and stroke: systematic review and meta-analysis. BMJ. 2015;24(350):h1295. 10.1136/bmj.h1295 PMC4373601 25810496

[8] Feigin VL, Roth GA, Naghavi M, Parmar P, Krishnamurthi R, Chugh S, et al Global burden of stroke and risk factors in 188 countries, during 1990–2013: a systematic analysis for the Global Burden of Disease Study 2013. Lancet Neurol. 2016;15(9):913–24. 10.1016/S1474-4422(16)30073-4 27291521

[9] Pope CA, Dockery DW. Health effects of fine particulate air pollution: lines that connect. J Air Waste Manag Assoc. 2006;56(6):709–42. 16805397 10.1080/10473289.2006.10464485

[10] Wang W, Jiang B, Sun H, Ru X, Sun D, Wang L, et al Prevalence, Incidence, and Mortality of Stroke in China: Results from a Nationwide Population-Based Survey of 480 687 Adults. Circulation. 2017;135(8):759–71. 28052979 10.1161/CIRCULATIONAHA.116.025250

[11] Kan H, Chen R, Tong S. Ambient air pollution, climate change, and population health in China. Environ Int. 2012;42:10–9. 10.1016/j.envint.2011.03.003 21440303

[12] Liu M, Huang Y, Ma Z, Jin Z, Liu X, Wang H, et al Spatial and temporal trends in the mortality burden of air pollution in China: 2004–2012. Environ Int. 2017;98:75–81. 10.1016/j.envint.2016.10.003 27745948 PMC5479577

[13] Huang F, Luo Y, Guo Y, Tao L, Xu Q, Wang C, et al Particulate Matter and Hospital Admissions for Stroke in Beijing, China: Modification Effects by Ambient Temperature. J Am Heart Assoc. 2016;5(7):003437. 10.1161/JAHA.116.003437 PMC5015380 27413045

[14] Liu H, Tian Y, Xu Y, Zhang J. Ambient Particulate Matter Concentrations and Hospitalization for Stroke in 26 Chinese Cities: A Case-Crossover Study. Stroke. 2017;48(8):2052–9. 28663508 10.1161/STROKEAHA.116.016482

[15] Huang F, Luo Y, Tan P, Xu Q, Tao L, Guo J, et al Gaseous Air Pollution and the Risk for Stroke Admissions: A Case-Crossover Study in Beijing, China. Int J Environ Res Public Health. 2017;14(2):189. 10.3390/ijerph14020189 PMC5334743 28216595

[16] Tian Y, Xiang X, Wu Y, Cao Y, Song J, Sun K, et al Fine Particulate Air Pollution and First Hospital Admissions for Ischemic Stroke in Beijing, China. 2017;7(1):3897. 10.1038/s41598-017-04312-5 PMC5478610 28634403

[17] Xiang H, Mertz KJ, Arena VC, Brink LL, Xu X, Bi Y, et al Estimation of short-term effects of air pollution on stroke hospital admissions in Wuhan, China. PLoS ONE. 2013;8(4):e61168 10.1371/journal.pone.0061168 23593421 PMC3625157

[18] Tian L, Qiu H, Pun VC, Ho KF, Chan CS, Yu IT. Carbon monoxide and stroke: A time series study of ambient air pollution and emergency hospitalizations. Int J Cardiol. 2015;201:4–9. 10.1016/j.ijcard.2015.07.099 26282452

[19] Meng Q, Fang H, Liu X, Yuan B, Xu J. Consolidating the social health insurance schemes in China: towards an equitable and efficient health system. Lancet. 2015;386(10002):1484–92. 10.1016/S0140-6736(15)00342-6 26466052

[20] Manila: WHO Regional Office for the Western Pacific. People’s Republic of China health system review. 2015 [cited 2018 Sep. 2]. http://www.searo.who.int/entity/asia_pacific_observatory/publications/hits/hit_china/en/

[21] Zhao B, Su Y, He S, Zhong M, Cui G. Evolution and comparative assessment of ambient air quality standards in China. J Integr Environ Sci. 2016;13(2–4):85–102.

[22] Chen R, Samoli E, Wong CM, Huang W, Wang Z, Chen B, et al Associations between short-term exposure to nitrogen dioxide and mortality in 17 Chinese cities: the China Air Pollution and Health Effects Study (CAPES). Environ Int. 2012;45:32–8. 10.1016/j.envint.2012.04.008 22572114

[23] Chen R, Kan H, Chen B, Huang W, Bai Z, Song G, et al Association of particulate air pollution with daily mortality: the China Air Pollution and Health Effects Study. Am J Epidemiol. 2012;175(11):1173–81. 10.1093/aje/kwr425 22510278

[24] Chen R, Yin P, Meng X, Liu C, Wang L, Xu X, et al Fine Particulate Air Pollution and Daily Mortality. A Nationwide Analysis in 272 Chinese Cities. Am J Respir Crit Care Med. 2017;196(1):73–81. 10.1164/rccm.201609-1862OC 28248546

[25] Yin P, Chen R, Wang L, Meng X, Liu C, Niu Y, et al Ambient Ozone Pollution and Daily Mortality: A Nationwide Study in 272 Chinese Cities. Environ Health Perspect. 2017;125(11):117006 10.1289/EHP1849 29212061 PMC5947936

[26] Yin P, He G, Fan M, Chiu KY, Liu C, Xue A, et al Particulate air pollution and mortality in 38 of China’s largest cities: time series analysis. BMJ. 2017;356:j667 10.1136/bmj.j667 28292780 PMC6287590

[27] Lanzinger S, Schneider A, Breitner S, Stafoggia M, Erzen I, Dostal M, et al Ultrafine and Fine Particles and Hospital Admissions in Central Europe. Results from the UFIREG Study. Am J Respir Crit Care Med. 2016;194(10):1233–41. 10.1164/rccm.201510-2042OC 27224452

[28] Dominici F, Peng RD, Bell ML, Pham L, McDermott A, Zeger SL, et al Fine particulate air pollution and hospital admission for cardiovascular and respiratory diseases. JAMA. 2006;295(10):1127–34. 10.1001/jama.295.10.1127 16522832 PMC3543154

[29] Peng RD, Chang HH, Bell ML, McDermott A, Zeger SL, Samet JM, et al Coarse particulate matter air pollution and hospital admissions for cardiovascular and respiratory diseases among Medicare patients. JAMA. 2008;299(18):2172–9. 10.1001/jama.299.18.2172 18477784 PMC3169813

[30] Liu C, Yin P, Chen R, Meng X, Wang L, Niu Y, et al Ambient carbon monoxide and cardiovascular mortality: a nationwide time-series analysis in 272 cities in China. Lancet Planet Health. 2018;2(1):e12–e8. 10.1016/S2542-5196(17)30181-X 29615203

[31] Wang L, Liu C, Meng X, Niu Y, Lin Z, Liu Y, et al Associations between short-term exposure to ambient sulfur dioxide and increased cause-specific mortality in 272 Chinese cities. Environ Int. 2018;117:33–9. 10.1016/j.envint.2018.04.019 29715611

[32] Almond D, Chen Y, Greenstone M, Hongbin L. Winter heating or clean air? Unintended impacts of China’s Huai river policy. American Economic Review. 2009;99 (2):184–90

[33] Ebenstein A, Fan M, Greenstone M, He G, Zhou M. New evidence on the impact of sustained exposure to air pollution on life expectancy from China’s Huai River Policy. Proc Natl Acad Sci U S A. 2017;114(39):10384–9. 10.1073/pnas.1616784114 28893980 PMC5625887

[34] Altman DG, Bland JM. Interaction revisited: the difference between two estimates. BMJ. 2003;326(7382):219 12543843 10.1136/bmj.326.7382.219 PMC1125071

[35] Wellenius GA, Schwartz J, Mittleman MA. Air pollution and hospital admissions for ischemic and hemorrhagic stroke among medicare beneficiaries. Stroke. 2005;36(12):2549–53. 16254223 10.1161/01.STR.0000189687.78760.47

[36] Larrieu S, Jusot JF, Blanchard M, Prouvost H, Declercq C, Fabre P, et al Short term effects of air pollution on hospitalizations for cardiovascular diseases in eight French cities: the PSAS program. Sci Total Environ. 2007;387(1–3):105–12. 10.1016/j.scitotenv.2007.07.025 17727917

[37] Barnett AG, Williams GM, Schwartz J, Best TL, Neller AH, Petroeschevsky AL, et al The effects of air pollution on hospitalizations for cardiovascular disease in elderly people in Australian and New Zealand cities. Environ Health Perspect. 2006;114(7):1018–23. 10.1289/ehp.8674 16835053 PMC1513338

[38] Chen R, Yin P, Meng X, Wang L, Liu C, Niu Y, et al Associations Between Ambient Nitrogen Dioxide and Daily Cause-specific Mortality: Evidence from 272 Chinese Cities. Epidemiology. 2018;29(4):482–9. 10.1097/EDE.0000000000000829 29621056

[39] Liu H, Tian Y, Xu Y, Huang Z, Huang C, Hu Y, et al Association between ambient air pollution and hospitalization for ischemic and hemorrhagic stroke in China: A multicity case-crossover study. Environ Pollut. 2017;230:234–41. 10.1016/j.envpol.2017.06.057 28654881

[40] Seaton A, Dennekamp M. Hypothesis: ill health associated with low concentrations of nitrogen dioxide—an effect of ultrafine particles? Thorax. 2003;58(12):1012–5. 10.1136/thorax.58.12.1012 14645960 PMC1746551

[41] Gao J, Wang K, Wang Y, Liu S, Zhu C, Hao J, et al Temporal-spatial characteristics and source apportionment of PM2.5 as well as its associated chemical species in the Beijing-Tianjin-Hebei region of China. Environ Pollut. 2018;233:714–24. 10.1016/j.envpol.2017.10.123 29126093

[42] Liu H, Tian Y, Xiang X, Juan J, Song J, Cao Y, et al Ambient Particulate Matter Concentrations and Hospital Admissions in 26 of China’s Largest Cities: A Case-Crossover Study. Epidemiology. 2018;29(5):649–57. 10.1097/EDE.0000000000000869 29870428

[43] Brook RD, Rajagopalan S, Pope CA 3rd, Brook JR, Bhatnagar A, Diez-Roux AV, et al Particulate matter air pollution and cardiovascular disease: An update to the scientific statement from the American Heart Association. Circulation. 2010;121(21):2331–78. 20458016 10.1161/CIR.0b013e3181dbece1

[44] Costa AF, Hoek G, Brunekreef B, Ponce de Leon AC. Air Pollution and Deaths among Elderly Residents of Sao Paulo, Brazil: An Analysis of Mortality Displacement. Environ Health Perspect. 2017;125(3):349–54. 10.1289/EHP98 27713111 PMC5332200

[45] Qian Z, He Q, Lin HM, Kong L, Bentley CM, Liu W, et al High temperatures enhanced acute mortality effects of ambient particle pollution in the "oven" city of Wuhan, China. Environ Health Perspect. 2008;116(9):1172–8. 10.1289/ehp.10847 18795159 PMC2535618

[46] Yang F, Tan J, Zhao Q, Du Z, He K, Ma Y, et al Characteristics of PM 2.5 speciation in representative megacities and across China. Atmos Chem Phys. 2011;11(11):5207–19.

[47] Goldman GT, Mulholland JA, Russell AG, Strickland MJ, Klein M, Waller LA, et al Impact of exposure measurement error in air pollution epidemiology: effect of error type in time-series studies. Environ Health. 2011;10(61):10–61. 21696612 10.1186/1476-069X-10-61 PMC3146396

[48] Turner RM, Omar RZ, Yang M, Goldstein H, Thompson SG. A multilevel model framework for meta-analysis of clinical trials with binary outcomes. Stat Med. 2000;19(24):3417–32. 11122505 10.1002/1097-0258(20001230)19:24<3417::aid-sim614>3.0.co;2-l

[49] Thompson S, Kaptoge S, White I, Wood A, Perry P, Danesh J. Statistical methods for the time-to-event analysis of individual participant data from multiple epidemiological studies. Int J Epidemiol. 2010;39(5):1345–59. 10.1093/ije/dyq063 20439481 PMC2972437

