# Supplementary material for: Association between ambient air pollution and daily hospital admissions for ischemic stroke: A nationwide time-series analysis
Source: PLoS Med. 2018 Oct 4;15(10):e1002668. doi: 10.1371/journal.pmed.1002668 (PMC6171821; doi:10.1371/journal.pmed.1002668)
Supplement: S1 Table — CI, confidence interval; IQR, interquartile range; PC, percentage change. (DOCX) [file pmed.1002668.s001.docx]

**S1 Table.** PC and 95% CI in daily hospital admissions for ischemic stroke associated with IQR increases in same-day PM_2.5_ (22.2 μg/m^3^), SO_2_ (17.5 μg/m^3^), NO_2_ (15.3 μg/m^3^), CO (0.41 mg/m^3^), and O_3_ (18.7 μg/m^3^) concentrations. CI, confidence interval; IQR, interquartile range; PC, percentage change.

| PM_2.5_ | SO_2_ | NO_2_ | CO | O_3_ |
| --- | --- | --- | --- | --- |
| 0.75 (0.44-1.07) | 2.53 (1.94-3.12) | 2.81 (2.23-3.40) | 1.39 (0.89-1.89) | 0 (-0.28-0.29) |
