# Supplementary material for: Association between ambient air pollution and daily hospital admissions for ischemic stroke: A nationwide time-series analysis
Source: PLoS Med. 2018 Oct 4;15(10):e1002668. doi: 10.1371/journal.pmed.1002668 (PMC6171821; doi:10.1371/journal.pmed.1002668)
Supplement: S2 Table — (DOCX) [file pmed.1002668.s002.docx]

**S2 Table.** Results of sensitivity analyses on the associations between air pollution (lag 0) and hospital admissions for ischemic stroke in 172 cities in China, 2014–2016.

| Variables | PM_2.5_ | SO_2_ | NO_2_ | CO |
| --- | --- | --- | --- | --- |
| Dataset |  |  |  |  |
| 73 cities with 2-year data | 0.59 (0.24-0.94) | 1.24 (0.55-1.93) | 2.11 (0.98-3.24) | 4.27 (1.65-6.96) |
| 99 cities with 3-year data | 0.27 (0.12-0.42) | 1.45 (1.07-1.83) | 1.81 (1.44-2.18) | 3.02 (1.67-4.39) |
| Degree of freedom for time |  |  |  |  |
| 6 | 0.35 (0.21-0.49) | 1.30 (0.98-1.61) | 1.78 (1.41-2.15) | 3.58 (2.34-4.83) |
| 7 | 0.34 (0.20-0.48) | 1.37 (1.05-1.70) | 1.82 (1.45-2.19) | 3.24 (2.05-4.43) |
| 8 | 0.34 (0.21-0.47) | 1.37 (1.05-1.70) | 1.76 (1.40-2.12) | 3.24 (2.15-4.34) |
| 9 | 0.34 (0.21-0.47) | 1.18 (0.87-1.48) | 1.69 (1.34-2.05) | 3.25 (2.17-4.35) |
| 10 | 0.31 (0.18-0.43) | 1.16 (0.85-1.46) | 1.71 (1.36-2.07) | 3.03 (1.94-4.12) |
| 11 | 0.32 (0.19-0.44) | 1.13 (0.83-1.42) | 1.65 (1.32-1.98) | 3.18 (2.13-4.24) |
| 12 | 0.31 (0.19-0.43) | 1.11 (0.77-1.33) | 1.59 (1.27-1.91) | 2.89 (1.83-3.95) |
| Degree of freedom for temperature |  |  |  |  |
| 4 | 0.30 (0.16-0.44) | 1.40 (1.07-1.73) | 1.78 (1.41-2.16) | 3.39 (2.15-4.64) |
| 5 | 0.30 (0.16-0.43) | 1.43 (1.10-1.76) | 1.81 (1.45-2.17) | 3.28 (2.08-4.48) |
| 6 | 0.29 (0.15-0.42) | 1.43 (1.10-1.75) | 1.79 (1.42-2.15) | 3.22 (2.02-4.44) |
| Degree of freedom for relative humidity |  |  |  |  |
| 4 | 0.34 (0.20-0.48) | 1.44 (1.10-1.77) | 1.83 (1.45-2.21) | 3.39 (2.15-4.64) |
| 5 | 0.34 (0.20-0.49) | 1.44 (1.11-1.77) | 1.83 (1.46-2.21) | 3.39 (2.15-4.64) |
| 6 | 0.35 (0.20-0.49) | 1.44 (1.10-1.77) | 1.83 (1.45-2.21) | 3.38 (2.14-4.63) |
