# Supplementary material for: Association between ambient air pollution and daily hospital admissions for ischemic stroke: A nationwide time-series analysis
Source: PLoS Med. 2018 Oct 4;15(10):e1002668. doi: 10.1371/journal.pmed.1002668 (PMC6171821; doi:10.1371/journal.pmed.1002668)
Supplement: S3 Table — CI, confidence interval; PC, percentage change. (DOCX) [file pmed.1002668.s003.docx]

**S3 Table.** PC and 95% CI in daily hospital admissions for ischemic stroke associated with increases of 10 μg/m^3^ in PM_2.5_, SO_2_, NO_2_, and O_3_ and 1 mg/m^3^ in CO concentrations (lag 0), classified by season. CI, confidence interval; PC, percentage change.

|  | PM_2.5_ | SO_2_ | NO_2_ | CO |
| --- | --- | --- | --- | --- |
| Warm season |  |  |  |  |
| South region | 0.61 (0.21-1.01) | 2.42 (1.16-3.70) | 2.51 (1.61-3.42) | 7.92 (3.90-12.11) |
| North region | 0.41 (0.15-0.67) | 0.79 (0.09-1.50) | 0.82 (0-1.65) | 1.98 (-0.02-4.01) |
| Cool season |  |  |  |  |
| South region | 0.33 (0.07-0.58) | 1.81 (1.00-2.62) | 2.27 (1.63-2.91) | 5.08 (2.59-7.63) |
| North region | -0.03 (-0.23-0.17) | 0.18 (-0.20-0.55) | 0.59 (0-1.17) | 0.33 (-1.30-1.99) |

Warm season: from April to September; Cool season: from October to March.
