# Supplementary material for: Association between ambient air pollution and daily hospital admissions for ischemic stroke: A nationwide time-series analysis
Source: PLoS Med. 2018 Oct 4;15(10):e1002668. doi: 10.1371/journal.pmed.1002668 (PMC6171821; doi:10.1371/journal.pmed.1002668)
Supplement: S4 Table — CI, confidence interval; PC, percentage change. (DOCX) [file pmed.1002668.s004.docx]

**S4 Table.** PC and 95% CI in daily hospital admissions for ischemic stroke associated with increases of 10 μg/m^3^ in PM_2.5_, SO_2_, NO_2_, and O_3_ and 1 mg/m^3^ in CO concentrations (lag 0), classified by city-specific annual average temperatures and air pollutant levels. CI, confidence interval; PC, percentage change.

|  | PM_2.5_ | SO_2_ | NO_2_ | CO |
| --- | --- | --- | --- | --- |
| Warm and high-polluted area | 0.47 (0.30-0.64) | 1.52 (0.60-2.44) | 2.07 (1.54-2.60) | 7.20 (4.27-10.22) |
| Cool and high-polluted area | 0.11 (-0.09 -0.32) | 0.54 (0.24-0.84) | 1.38 (0.91-1.85) | 0.63 (-0.99-2.27) |
| Warm and low-polluted area | 0.76 (0.07-1.45) | 3.31 (2.16-4.48) | 4.20 (2.50-5.94) | 5.55 (2.15-9.06) |
| Cool and low-polluted area | 0.63 (0.21-1.05) | 2.11 (0.20-4.05) | 0.92(-0.52-2.38) | 4.92 (1.26-8.70) |

Warm and high-polluted area: area with temperature > 50th (16.4 °C) and air pollutants levels > 50th; Warm and low-polluted area: area with temperature > 50th and air pollutants levels ≤ 50th; Cool and high-polluted area: area with temperature ≤ 50th and air pollutants levels > 50th; Cool and low-polluted area: area with temperature ≤ 50th and air pollutants levels ≤ 50th.
